# Remarks upon Codeia

**Published:** 1838-12-19

**Authors:** Joseph Carson

**Affiliations:** Professor of Materia Medica in the College of Pharmacy


Remarks upon Codeia. By Joseph Carson, M. D., *Professor of Materia Medica in the College of Pharmacy.*

Several years have now elapsed since the discovery by Robiquet of this proximate principle in opium; but it seems to have been regarded by the profession generally, rather as a subject of curiosity, evincing the accuracy and minuteness to which chemical research has been carried, than as an agent capable of therapeutic application. In this country, at least, the article is comparatively little known, its powers over the animal economy having been overlooked,—or it has been ranked with the host of new remedies, which have in succession presented their claims to notoriety, but which, when tried, have been found not to possess the value that had been attributed to them. Repeated observation, however, of the effects of codeia by a few European practitioners, has tended to demonstrate that it is worthy of attention as a medicine adapted to particular conditions of disease, and perhaps solely calculated to fulfil indications that otherwise might baffle the most skilful. Upon comparing the reports that have been made, it will be found that in each nearly the same properties have been attributed to it, and that these are among the most efficient with which a substance can be endowed. The individuals who have made it an object of experiment, are Messrs. Kunkel and Barbier, whose results were published in the Revue Medicale for the year 1833, and the Journal de Pharmacie for 1834, and republished in the 6th volume of the American Journal of Pharmacy. The subsequent experience of M. Barbier has confirmed him in the conclusions drawn from his first experiments; for in the last edition of his valuable Treatise upon the Materia Medica, just published, he has introduced it as an addition to the list of narcotics, leaving to pharmaceutic writers the question as respects the propriety of the name *codeina*, which he has bestowed upon it. We shall present to the readers of this journal a condensed account of the substance and its properties, derived from the source above specified.

Codeia is a whitish crystallizable substance, soluble in water, and alcohol. It is distinguished from morphia by its solubility in ether,—from which also it differs in not being dissolved by the caustic alkalies, and in not decomposing the salts of the protoxide of iron. It combines with acids, and exists in opium, in combination with meconic acid. Nitric acid does not communicate to it a red colour.

Codeia is administered in the dose of two grains. It can be made into pills by means of conserve of roses, or any other excipient. M. Barbier administers it generally in syrup, the strength of which is two grains to the ounce. This syrup may be employed to form other preparations.

It appears, from the investigations of M. Barbier, that, in consequence of the high price of the substance, gross imposition was practised by apothecaries when it first came into notice in France, and that those who were induced to try its remedial effects, were disappointed from this circumstance, the acetate and muriate of morphia having been substituted for it. The taste is bitter and disagreeable; it produces no immediate effect when taken into the stomach, sometimes, perhaps, a sensation of warmth. It does not affect digestion, or disturb the appetite, and does not, like opium, produce constipation. M. Barbier is of opinion that the power of codeia is especially directed to the plexus of ganglionic nerves, and that it also produces a modification of the condition of the cerebral hemispheres, essentially different, however, from that occasioned by morphia.

As regards the kind of action that codeia produces in the ganglionic plexus, or the character of the change effected in the centres of innervation, we possess little knowledge. These are points to be settled when pathology shall have enlightened physiology upon the part they play in health and disease; but, at the same time, it cannot be denied that this nervous apparatus is of vital importance, that its force is liable to variation from depression or augmentation, that disturbance of its operation occurs, and that particular diseased conditions result, over which codeia may have a salutary control; reproducing the normal state, and with the removal of the perturbatory influence, dissipating the attendant symptoms, at times distressing, if not alarming. The conditions referred to, are manifested by weight and oppression below the sternum, vague and diffuse pain in the epigastrium, extending to the sides and back, palpitation of the heart, difficulty of respiration, dry cough, spasms of the diaphragm, pulsation of the abdomen, and even vomiting, without loss of appetite, or deranged digestion; if still more violent, the face becomes pale and its expression anxious, the pulse is irregular, chilliness and profuse perspiration rapidly succeed each other, and the various moral emotions are awakened, as grief, terror, &c. Now, although there is no uniformity in all these symptoms, yet, in order to explain them, it is necessary to regard them as emanating from a certain morbid condition of the epigastric plexus, and floating through the irradiations of the plexus which embrace the thoracic and abdominal viscera, without appertaining to any particular organ; hence nosology has neither rank or name for the affection. Codeia, if not a specific in this case, is of positive value. That it exerts influence especially upon the ganglionic system of nerves, is further strengthened by the anomalous symptoms to which it sometimes gives rise in individuals of great nervous susceptibility, as pain in the chest and belly, nausea and vomiting, and threatened syncope; these are nevertheless exceptions.

As respects its influence upon the cerebral hemispheres, there is but a single analogy between it and morphia, and this is that they both produce sleep; the sleep, however, is dissimilar, that from morphia is heavy, disturbed by reveries, with puffiness of the features, falling of the eyelids, and dull, inexpressive appearance of the eyes; when roused from such sleep, there is confusion of thought and stupidity. But, on the contrary, after the exhibition of codeia, the sleep is light and refreshing, the individual is readily roused, the eyes are animated and brilliant, the lids are under control, the features are calm, and can be readily excited. Morphia tends to produce sanguine congestion in the vessels of the cerebrum, which is not the case with codeia; in fact, the mode of operation is so unlike that from outward appearances, that M. Barbier states he is confident he can determine which of the two substances has been taken.

It does not appear that any power is possessed by it over the spinal marrow.

The therapeutic application of codeia, where reasons exist to prevent the exhibition of opium or morphia in diseases of the brain, may be learnt from the following case, reported by M. Barbier.

A young man, in the Hôtel Dieu of Amiens, labored under a marked affection of the head, he lost entirely the power to sleep. For six weeks he passed the nights in unconquerable insomnolency. Opium, and the salts of morphia, only caused frightful reveries and painful drowsiness, but no sleep. The syrup of codeia procured for him, the first day it was administered, five hours of calm refreshing rest.

The same advantages are secured by endermic application as from morphia.

If further evidence be required, the testimony of Dr. Miranda, of Havana, may be adduced, to show that codeia has a degree of efficiency not possessed by other remedies. He has announced the cure of eleven cases of an affection, which has been denominated by him *gastritis*, some of which had resisted long medication by different means. The medicine was exhibited for a month or more, and the quantity of syrup taken in the twenty-four hours did not exceed six drachms.*

With the recommendation proceeding from the source above cited, it appears to us that practitioners should not delay the trial of a remedial agent which holds out so much promise of advantage. Cases constantly are presenting themselves in every way fitted for experimental exhibition, the result of which should be carefully noted and recorded. With this view it is that the present notice has been penned.

The article is manufactured by Mr. Rosengarten, an enterprising and accomplished chemist of Philadelphia. The muriate has also been prepared by him.

* The following is the formula for the syrup of codeia:—

R.—Codeia, gr. xxiv.

Distilled water, ℥iv.

White sugar, ℥viij.

Reduce the codeia to an impalpable powder; triturate it with a third of the water, allow it to settle and decant. Act upon the residuum with another third of the water, and then again with the remainder. Reunite the whole in a small matrass, covering the opening with a piece of moistened parchment, pierced with a pin-hole. Heat in a salt-water bath until the codeia has entirely disappeared, remove the matrass from the fire to add the sugar, eover the opening anew, agitate it, plunge it again into the bath, and allow it to remain until the sugar is completely dissolved. Filter in a cold situation, and preserve it by the ordinary means.

